# Degenerative cervical myelopathy

**DOI:** 10.1136/bmj.k186

**Published:** 2018-02-22

**Authors:** Benjamin M Davies, Oliver D Mowforth, Emma K Smith, Mark RN Kotter

**Affiliations:** 1 Academic neurosurgery unit, Department of Clinical Neurosurgery, University of Cambridge, Cambridge, UK; 2 School of General Practice, NHS Health Education East of England, UK

**What you need to know**

Consider degenerative cervical myelopathy in patients over 50 with progressive neurological symptoms, such as pain and stiffness in the neck or limbs, imbalance, numbness, loss of dexterity, frequent falls, and/or incontinence
Perform a full neurological assessment as early symptoms are subtle and non-specific
A magnetic resonance imaging (MRI) scan is essential to detect degenerative changes in the cervical spine and cord compression
**Time is Spine:** Refer patients with suspected DCM promptly to a specialist for consideration of spinal surgery, as delayed diagnosis can lead to residual symptoms and functional disability

A 54 year old man presents with neck stiffness for about a year. He complains of numbness in his fingers and difficulty buttoning up his shirt, which has not improved following surgery for carpal tunnel syndrome. Of late, he has experienced unsteadiness and has started to use a walking stick after sustaining falls. He sees a neurologist who identifies hyperreflexia in his arms and legs. An MRI scan shows multilevel cervical spondylosis and disc herniation causing cord compression. He is diagnosed with degenerative cervical myelopathy and referred to spinal surgery for operative decompression.

## What is degenerative cervical myelopathy?

**Fig 1:**
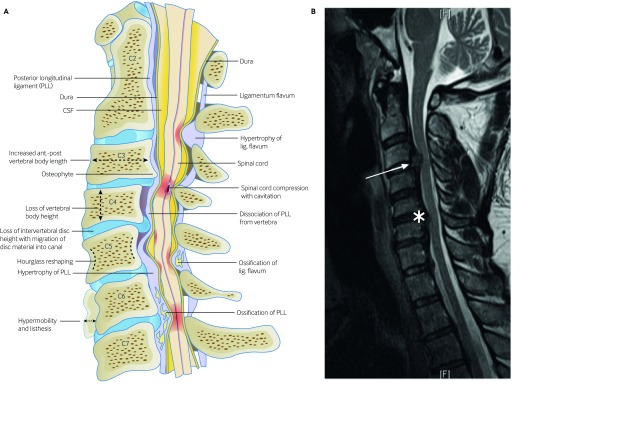
Pathology of DCM. (A) Anatomy of an initially healthy spine (C2 level), with examples of the potential pathological changes that can occur and cause DCM (shown at lower spinal levels; C3-7).^1^ (B) Sagittal section from a T2-weighted MRI scan showing multilevel degenerative changes in the cervical spine. The spinal cord is compressed at C3/4 by a disc prolapse (white arrow) and at C5/6 by spondylosis, thickening of the posterior longitudinal ligament, and a disc-osteophyte complex (white star). However, this is not associated with high signal changes in the cord on MRI (Figure reproduced with permission from Michael G Fehlings, University of Toronto)^3^

Degenerative cervical myelopathy (DCM), earlier referred to as cervical spondylotic myelopathy, involves spinal cord dysfunction from compression in the neck.^1^ Patients report neurological symptoms such as pain and numbness in limbs, poor coordination, imbalance, and bladder problems. Owing to its mobility, the vertebral column of the neck is particularly prone to degenerative changes such as disc herniation, ligament hypertrophy or ossification, and osteophyte formation. These changes are more common with age^2^ (box 1) and are often collectively termed *spondylosis* (fig 1).^3^

Box 1 **How common is it?**

The epidemiology of DCM is poorly understood, in part because of the difficulties in diagnosis.^3^

- The prevalence of surgically treated DCM is estimated as 1.6 per 100 000 inhabitants.^4^ The actual prevalence is likely to be much higher
- The incidence of DCM is expected to rise with an ageing population.^2^ ^3^ Most patients are first diagnosed in their 50s; DCM is uncommon before the age of 40
- Studies in healthy volunteers have shown that incidental cervical cord compression is commonly detected on MRI, and becomes more common with age.^5^ ^6^ In a series of randomly selected volunteers aged 40-80, incidental cervical cord compression was detected on MRI in 59% of individuals (108/183, ranging from 31.6% in the fifth decade to 66.8% in the eighth decade). Only two individuals reported related symptoms^2^
- A proportion of individuals with asymptomatic cord compression will go on to develop DCM. The exact figure is unknown. The only prospective study to consider this (n=199) found that 8% of individuals with asymptomatic cord compression will develop DCM after one year and 22% in total over the observation period (median follow-up 44 months, range 2-12 years)^7^
- Many patients with DCM remain undiagnosed. A small study in 66 patients with hip fracture found 18% of patients who were previously undiagnosed to have clinical findings suggestive of DCM^8^

## Why is it missed?

Non-specific and subtle early features that overlap with other neurological conditions can delay the diagnosis.^9^ Incomplete neurological assessment by professionals^10^ with a poor awareness of the disease^11^ further contributes to delay. A retrospective study of medical records of 42 patients in Israel who underwent surgery for DCM noted an average delay of 2.2 years (range 1.7 months to 8.9 years) from initiation of symptoms to diagnosis. On average, 5.2 ±3.6 consultations were required before a diagnosis was made.^10^ Forty three per cent of these patients had symptoms of numbness and pain in hands, and were initially diagnosed and sometimes treated for carpal tunnel syndrome.^10^ In our clinical experience, the diagnosis of carpal tunnel syndrome, especially when diagnosed bilaterally, is often incorrect and DCM usually accounts for these symptoms.

## Why does this matter?

Spinal cord compression results in progressive neurological decline and affects quality of life.^12^ Left untreated, it can lead to tetraplegia and wheelchair dependence (data on how many patients with DCM progress in this way are unavailable). Surgical decompression can halt the disease progression, however, the regenerative capacity of the spinal cord is limited and any damage is often permanent. Delayed treatment leads to poorer outcomes and lifelong disability. Findings from the AOSpine series (746 patients with DCM) indicate that treatment within six months of symptoms offers the best chance of recovery,^13^ but this time frame is some way from current average diagnosis times.^10^

## How is it diagnosed?

Detecting early DCM can be challenging. A high index of suspicion, alongside a comprehensive neurological examination is advised. Box 2 outlines common symptoms and examination findings in DCM.

Box 2 **Commonly reported symptoms and examination findings in DCM^9^**

**Symptoms**

- Neck pain/stiffness
- Unilateral or bilateral limb/body pain
- Upper limb weakness, numbness, or loss of dexterity
- Lower limb stiffness, weakness, or sensory loss
- Paraesthesia (tingling or pins and needles sensations)
- Autonomic symptoms such as bowel or bladder incontinence, erectile dysfunction, or difficulty passing urine
- Imbalance/unsteadiness
- Falls

**Examination findings**

- Motor signs
  o Pyramidal weakness (Upper limb; extensors more than flexors. Lower limb: flexors more than extensors)
  o Limb hyperreflexia
  o Spasticity (eg, clasp knife sign)
  o Clonus, especially Achilles tendon
  o Hoffman’s sign (thumb adduction/flexion +/− finger flexion after forced flexion and sudden release of a finger, distally)
  o Babinski’s sign (upgoing plantar)
  o Segmental weakness (corresponding to the level of compression)
- Sensory loss (limb and/or trunk)
- Lhermitte’s sign (electric shock sensation down the spine, or into the limbs, on neck flexion or extension, present in severe cases)
- Gait disturbance

### Clinical

Pain is a common reason to seek treatment. Musculoskeletal pain might be present in the neck, while neuropathic pain can affect upper and lower limbs and occasionally the trunk. Patients often report neck stiffness, at times without pain. A textbook case would describe gait dysfunction and bilateral hand impairment. Frequently not all symptoms are present. For example, pain might be absent and symptoms can be unilateral and vary in severity, even on a daily basis.^9^ Atypical symptoms such as headaches and muscle cramps are also reported.^9^

The more consistent feature of DCM is the evolution of symptoms. Most patients describe symptoms that have been ongoing for months and getting worse.^9^ The rate of progression varies; in some individuals symptoms remain mild over extended periods of time, while in others disease progression accelerates. Functional decline can be insidious, and patients might mistakenly attribute these symptoms to “getting older.” Typical features include loss of dexterity (difficulty doing up buttons, using keys, mobile phones, or writing) or mobility (use of walking aids or frequent falls).

Symptoms might precede objective examination findings.^9^ ^14^ As in focal central nervous system disorders, examination features in DCM have a low sensitivity—that is, a normal finding does not exclude the disease— but high specificity—that is, an abnormal finding is highly suggestive of the disease.^5^ ^14^ Features can be mild and difficult to elicit in the initial stages of disease.

### Investigations

Request an MRI scan of the cervical spine to detect cord compression (fig 1) in suspected DCM. An urgent MRI is required for patients with progressive disease and/or symptoms that substantially affect quality of life. In patients with mild symptoms, a non-urgent MRI might be requested. Bear in mind that the extent of spinal cord compression and signal changes in the cord on the MRI scan do not correlate well with the severity of symptoms.^3^ Even mild compression can account for severe disease.

The pathway to diagnosis varies depending on local services. In the UK, for example, many primary care physicians do not have direct access to MRI imaging and referral to neurology might be warranted.

## How is it managed?

Often cord compression is an incidental finding and at least initially does not cause symptoms.^2^ Reassure the patient that no further management is required at this stage but advise them to report any symptoms promptly in the future.

Guidelines from AOSpine^1^an international community of spine surgeons advise that all patients with DCM should be assessed by a specialist surgeon, who might fall under the remit of neurosurgery or orthopaedics. The guidelines use the modified Japanese Orthopaedic Association score, which classifies patients as having mild or severe symptoms based on arm, leg, and bladder function.^1^ Surgery is recommended in patients with moderate or severe DCM and in those with disease progression. Treatment of symptoms (for pain, for example) and regular follow-up might be offered for patients with mild, stable DCM.

The AOSpine series showed that decompressive surgery can halt disease progression and enable meaningful, albeit limited, recovery across a range of measures including pain, function, and quality of life.^15^ The optimal timing of surgery is debatable because the progression of disease is poorly understood.^9^ Preoperative physiotherapy should only be advised by specialist services^1^; neck manipulation is strictly contraindicated as it might cause further damage.^16^

It is not possible to predict the long term outcome of surgery. Maximal recovery occurs at around 6-12 months. Residual symptoms beyond this are likely to be permanent and should be managed appropriately. Functional deficits are common, and include falls and reduced mobility, incontinence, depression, sleep deficits, and struggles with self-care, and often the most troublesome symptom is pain. Discuss with your patient that complete resolution of pain is unlikely. Neuropathic analgesia and anti-spasticity medication can be offered to manage the pain. Early referral to specialist pain clinics is often helpful.

Ask patients to report any worsening or new symptoms or signs as untreated levels of the cervical spine might further degenerate and cause spinal cord compression.

**Education into practice**

What features would prompt you to suspect DCM in a patient?

How would you explain a diagnosis of DCM to your patient?

Are you aware of the appropriate local pathways for arranging an urgent MRI scan for patients with suspected DCM?

After reading this article, are there any aspects of imaging or referral that you would approach differently?

**How patients were involved in the creation of this article**

This article was reviewed and endorsed by individuals experiencing DCM who were part of a committee at Myelopathy.org. The committee was keen to emphasise the possible prevalence of DCM and its long term effects, even after surgery. More specifically, it was involved in shaping the paragraph “What is DCM?”

Myelopathy.org (www.myelopathy.org) is the first organisation dedicated to raising awareness, providing information, and supporting research for DCM. It provides a forum for individuals to communicate their experiences of DCM and offers peer to peer support to patients. Reports of delayed and/or misdiagnosis are common, which result from a lack of awareness among frontline medical specialties, particularly primary care. The committee proposed an educational initiative, which included this article.

## Notes

Acknowledgments: Research in the senior author’s laboratory is supported by a core support grant from the Wellcome Trust and MRC to the Wellcome Trust-Medical Research Council Cambridge Stem Cell Institute. MRNK is supported by a NIHR Clinician Scientist Award.

Disclaimer: This report is independent research arising from a Clinician Scientist Award, CS-2015-15-023, supported by the National Institute for Health Research. The views expressed in this publication are those of the authors and not necessarily those of the NHS, the National Institute for Health Research or the Department of Health.

All authors have contributed to the development, drafting and revision of this article and agree to its submission.

Provenance and peer review: commissioned; externally peer reviewed.

## References

[1] Fehlings MG Tetreault LA Riew KD A clinical practice guideline for the management of patients with degenerative cervical myelopathy: recommendations for patients with mild, moderate, and severe disease and nonmyelopathic patients with evidence of cord compression. Global Spine J 2017;7(Suppl):70S-83S. 10.1177/2192568217701914 29164035 PMC5684840

[2] Kovalova I Kerkovsky M Kadanka Z Prevalence and imaging characteristics of non-myelopathic and myelopathic spondylotic cervical cord compression. Spine (Phila Pa 1976) 2016;41:1908-16. 10.1097/BRS.0000000000001842 27509189

[3] Nouri A Tetreault L Singh A Karadimas SK Fehlings MG Degenerative cervical myelopathy: epidemiology, genetics, and pathogenesis. Spine (Phila Pa 1976) 2015;40:E675-93. 10.1097/BRS.0000000000000913 25839387

[4] Boogaarts HD Bartels RHMA Prevalence of cervical spondylotic myelopathy. Eur Spine J 2015;24(Suppl 2):139-41. 10.1007/s00586-013-2781-x 23616201

[5] Nagata K Yoshimura N Muraki S Prevalence of cervical cord compression and its association with physical performance in a population-based cohort in Japan: the Wakayama Spine Study. Spine (Phila Pa 1976) 2012;37:1892-8. 10.1097/BRS.0b013e31825a2619 22565382

[6] Okada E Matsumoto M Ichihara D Aging of the cervical spine in healthy volunteers: a 10-year longitudinal magnetic resonance imaging study. Spine (Phila Pa 1976) 2009;34:706-12. 10.1097/BRS.0b013e31819c2003 19333104

[7] Bednarik J Kadanka Z Dusek L Presymptomatic spondylotic cervical myelopathy: an updated predictive model. Eur Spine J 2008;17:421-31. 10.1007/s00586-008-0585-1 18193301 PMC2270386

[8] Radcliff KE Curry EP Trimba R High incidence of undiagnosed cervical myelopathy in patients with hip fracture compared to controls. J Orthop Trauma 2016;30:189-93. 10.1097/BOT.0000000000000485 26562581

[9] Tracy JA Bartleson JD Cervical spondylotic myelopathy. Neurologist 2010;16:176-87. 10.1097/NRL.0b013e3181da3a29 20445427

[10] Behrbalk E Salame K Regev GJ Keynan O Boszczyk B Lidar Z Delayed diagnosis of cervical spondylotic myelopathy by primary care physicians. Neurosurg Focus 2013;35:E1. 10.3171/2013.3.FOCUS1374 23815245

[11] Roberts E. Elemental ideas: cervical spondylotic myelopathy. www.myelopathy.org

[12] Oh T Lafage R Lafage V Comparing quality of life in cervical spondylotic myelopathy with other chronic debilitating diseases using the SF-36 survey. World Neurosurg 2017;106:699-706. 10.1016/j.wneu.2016.12.124 28065875

[13] Tetreault LA Côté P Kopjar B Arnold P Fehlings MG AOSpine North America and International Clinical Trial Research Network A clinical prediction model to assess surgical outcome in patients with cervical spondylotic myelopathy: internal and external validations using the prospective multicenter AOSpine North American and international datasets of 743 patients. Spine J 2015;15:388-97. 10.1016/j.spinee.2014.12.145 25549860

[14] Nicholl DJ Appleton JP Clinical neurology: why this still matters in the 21st century. J Neurol Neurosurg Psychiatry 2015;86:229-33. 10.1136/jnnp-2013-306881 24879832 PMC4316836

[15] Fehlings MG Ibrahim A Tetreault L A global perspective on the outcomes of surgical decompression in patients with cervical spondylotic myelopathy: results from the prospective multicenter AOSpine international study on 479 patients. Spine (Phila Pa 1976) 2015;40:1322-8. 10.1097/BRS.0000000000000988 26020847

[16] Rhee JM Shamji MF Erwin WM Nonoperative management of cervical myelopathy: a systematic review. Spine (Phila Pa 1976) 2013;38(Suppl 1):S55-67. 10.1097/BRS.0b013e3182a7f41d 23963006

